# Changes in Hepcidin Levels in an Animal Model of Anemia of Chronic Inflammation: Mechanistic Insights Related to Iron Supplementation and Hepcidin Regulation

**DOI:** 10.1155/2021/4357756

**Published:** 2021-11-27

**Authors:** Hye-Bin Kim, Ji Hae Jun, Jae-Kwang Shim, Ju Eun Oh, Cheolhun Lee, Young-Lan Kwak

**Affiliations:** ^1^Department of Anesthesiology and Pain Medicine, Anesthesia and Pain Research Institute, Yonsei University College of Medicine, Seoul 03722, Republic of Korea; ^2^Department of Anesthesiology and Pain Medicine, Yonsei University College of Medicine, Seoul 03722, Republic of Korea

## Abstract

We examined changes in hepcidin (closely associated with anemia of chronic inflammation (ACI)) and upstream regulatory pathways after intravenous (IV) iron supplementation in an ACI animal model. ACI was induced in male Sprague-Dawley rats by intraperitoneally administering complete Freund's adjuvant (CFA). Two weeks after starting CFA treatment, ACI rats received IV iron (CFA-iron) or vehicle (CFA-saline). Three days after IV iron treatment, iron profiles, hepcidin levels, and expression of proteins involved in the signaling pathways upstream of hepcidin transcription in the liver were measured. In CFA-treated rats, anemia with a concomitant increase in the levels of serum inflammatory cytokines and reactive oxygen species occurred. In CFA-iron rats, hemoglobin (Hb) concentration was still lower than that in control rats. In CFA-saline rats, hepatic hepcidin and ferritin levels increased compared with those in control rats and were further increased in CFA-iron rats. In CFA-saline rats, NADPH oxidase- (NOX-) 2, NOX-4, and superoxide dismutase levels in the liver were upregulated compared with those in control rats and their levels were further increased in CFA-iron rats. In CFA-saline rats, activities of the IL-6/STAT and BMP/SMAD pathways were enhanced in the liver compared with those in control rats and their levels were further increased in CFA-iron rats, whereas IL-6 expression remained unaffected after IV iron administration. In HepG2 cells, iron caused phosphorylation of STAT-3 and SMAD1/5 and knockdown of *STAT-3* and *SMAD1/5* using *siRNA*s reduced iron-induced hepcidin upregulation to levels similar to those in corresponding control cells. Renal erythropoietin expression and serum erythroferrone concentration were lower in CFA-iron rats than those in control rats. In ACI rats, IV iron supplementation did not recover Hb within three days despite an increase in hepatic ferritin levels, which might be attributable to an additional increase in hepcidin levels that was already upregulated under ACI conditions. Both STAT-3 phosphorylation and SMAD1/5 phosphorylation were associated with hepcidin upregulation after IV iron treatment, and this seems to be linked to iron-induced oxidative stress.

## 1. Introduction

Anemia of chronic inflammation (ACI), also referred to as anemia of chronic disease, is the second most common type of anemia after iron deficiency anemia (IDA) in hospitalized patients and is associated with increased morbidity [1, 2]. Inadequate utilization of iron despite sufficient body iron stores leads to the development of ACI, whereas IDA is caused by an absolute iron deficiency [3].

Hepcidin is a key regulator of iron homeostasis and is known to regulate iron metabolism through ferroportin (FPN), the sole known iron exporter [4, 5]. Hepcidin is upregulated by an increase in iron stores in the body and inflammatory conditions and is downregulated by erythropoiesis [4, 5]. Iron-mediated regulation of hepcidin levels acts mainly through the bone morphogenetic protein (BMP)/Sma mothers against the decapentaplegic (SMAD) signaling pathway. Inflammation and reactive oxygen species (ROS) also influence the expression of hepcidin, mainly by triggering the signal transducer and activator of the transcription-3 (STAT-3) pathway [6]. By contrast, erythroferrone (ERFE), secreted by erythropoietin- (EPO-) stimulated erythroblasts during erythropoiesis, suppresses the expression of hepcidin [7, 8]. Considering the increased hepcidin expression and the resultant decrease in FPN expression and iron sequestration in iron storage cells in ACI, the hepcidin/FPN axis is an ideal target for the treatment of ACI [9]. However, currently there is no drug available that can directly regulate this axis. Iron supplementation and blood transfusion are the only clinical treatment regimens for iron deficiency and anemia, regardless of the underlying mechanisms.

In the current clinical practice, intravenous (IV) administration of iron is preferred over oral administration because of its superior efficacy in terms of bioavailability and simple administration protocol [10, 11]. Furthermore, the absorption of dietary iron through duodenal enterocytes decreases as hepcidin expression increases. The stable dextran-based iron complex, containing minimal amount of labile iron, is well tolerated by patients even at high doses during IV administration because of its robust and reliable pharmacokinetic properties [12, 13]. Theoretically, however, iron has two opposite effects on erythropoiesis: it activates erythroid differentiation directly but inhibits it indirectly by stimulating hepcidin and enhancing oxidative stress [14]. In this context, recent clinical studies reported various results related to the impact of a high-dose IV iron on hemoglobin (Hb) levels, especially during a short period of time [15–18]. However, there is a paucity of data on changes in hepcidin levels in relation to IV iron supplementation under ACI conditions in both experimental and clinical studies.

Therefore, the primary aim of this study was at investigating short-term changes in hepcidin expression and its regulatory pathways in the liver after IV iron supplementation at a clinically used concentration in a relevant animal model of ACI, which resembles its clinical presentation. The secondary aim of this study was at evaluating the concomitant changes in renal *Epo* expression and serum ERFE concentration, as well as oxidative stress markers, after IV iron supplementation. We also aimed to identify the role of the signaling pathways upstream to hepcidin in HepG2 cells loaded with iron using short interfering RNAs.

## 2. Materials and Methods

### 2.1. Animal Preparation

All animal procedures were approved by the Committee for the Care and Use of Laboratory Animals, Yonsei University College of Medicine, and were performed in accordance with the Guide for the Care and Use of Laboratory Animals published by the National Institutes of Health (USA). Male Sprague-Dawley rats (130−150 g) were housed in a temperature-controlled environment at 23–25°C with a 12/12 h light/dark cycle and were provided *ad libitum* access to food and water.

### 2.2. Study Groups and Experimental Models

Animals were randomly assigned to three groups: control-saline (*n* = 16), complete Freund's adjuvant- (CFA-) saline (InvivoGen, San Diego, CA, USA) (*n* = 16), and CFA-iron (iron isomaltoside, Monofer®; Pharmacosmos A/S, Holbaek, Denmark; *n* = 10).

For the establishment of the ACI model, rats were intraperitoneally (IP) administered 0.2 mL of CFA containing 1 mg/mL of heat-killed *Mycobacterium tuberculosis* mixed with phosphate buffer saline (in the ratio of 1 : 1, thrice across two weeks), whereas the control rats were administered the same volume of saline [19]. Rats that exhibited a decline in Hb by >2 g/dL from baseline after two weeks were designated as ACI rats [20]. ACI rats received 20 mg/kg of IV iron (which is the highest clinical dose currently used) or an equivalent volume of saline. Serum and tissues were harvested from euthanized rats three days after IV iron or saline administration.

### 2.3. Hematologic Studies

Blood samples were subjected to complete blood counts obtained using a Mindray BC-5000 Vet hematology analyzer (Shenzhen Mindray Bio-Medical Electronics, Shenzhen, China).

### 2.4. Measurement of Iron Parameters

Transferrin saturation (TSAT) and total iron-binding capacity (TIBC) were analyzed by an automated chemistry analyzer (Cobas C702; Roche, Mannheim, Germany) at Seoul Clinical Laboratories (Seoul, Korea).

### 2.5. Immunohistochemistry (IHC)

Liver tissue samples were subjected to IHC analysis. The samples were washed in physiological saline, fixed in 10% buffered formalin, and embedded in paraffin. Sections were then stained with rabbit anti-NADPH oxidase- (NOX-) 2 (1 : 100, Novus, Littleton, CO, USA) and anti-NOX-4 (1 : 500, Abcam, Cambridge, MA, USA), followed by staining with chromogen 3,3-diaminobenzidine (Abcam). Slides were viewed with an Olympus IX73P2F microscope (Olympus America, Melville, NY, USA) equipped with an Olympus DP71 digital camera (20×).

### 2.6. Cell Culture

HepG2 cells (human liver cancer cell line) were purchased from the American Type Culture Collection (Rockville, MD, USA) and were maintained in Eagle's minimal essential medium (EMEM) + 2 mM glutamine supplemented with 10% foetal bovine serum (FBS), 100 unit/mL penicillin, and 100 *μ*g/mL streptomycin at 37°C in a humidified atmosphere containing 95% air and 5% CO_2_. Culture media and supplements were purchased from Gibco (Carlsbad, CA, USA).

### 2.7. Cell Viability Assay

**Figure 1:**
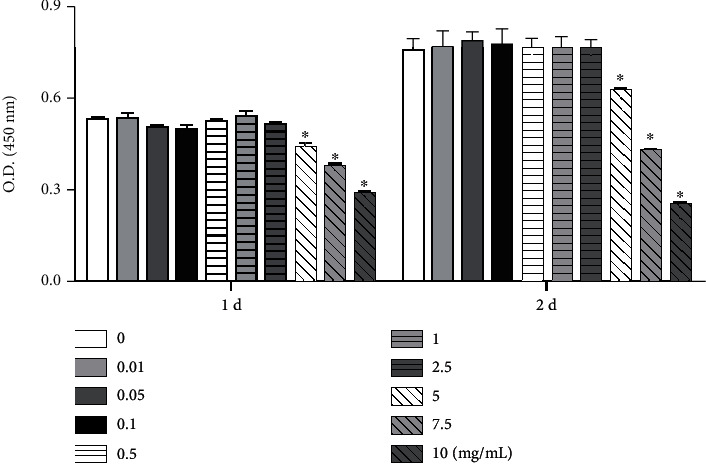
Cell viability of HepG2 cells treated with varying concentrations of iron. Cells were incubated for one or two days in the presence of iron at the concentrations indicated (0−10 mg/mL), and then, the number of viable cells was analyzed using the Cell Counting Kit-8 assay. O.D.: optical density. ^∗^*P* < 0.05 compared with the control group.

HepG2 cells (5 × 10^3^) were seeded in 96-well culture plates and incubated overnight. The cells were then incubated for 0–2 days in EMEM + 2 mM glutamine supplemented with 10% FBS with or without iron, at the concentrations indicated (Figure 1). Finally, cell viability was evaluated using the Cell Counting Kit- (CCK-) 8 assay (Dojindo, Kumamoto, Japan) according to the manufacturer's protocol. Experiments were performed in triplicate.

### 2.8. Immunoblot Analysis

Tissue specimens were lysed in radioimmunoprecipitation assay buffer supplemented with 20 mM Tris-HCl (pH 7.5), 150 mM NaCl, 1 mM Na_2_EDTA, 1 mM EGTA, 1% Triton, 2.5 mM sodium pyrophosphate, a protease inhibitor mixture, and phosphatase inhibitor cocktail-2 and cocktail-3 (Sigma-Aldrich, St. Louis, MO, USA). Protein concentrations were measured using the Bradford assay (Thermo Fisher Scientific, Carlsbad, CA, USA), and equal amounts of protein from each sample were subjected to immunoblot assay. Proteins were separated using sodium dodecyl sulfate-polyacrylamide gel electrophoresis and immunoblotted with anti-hepcidin, anti-ferritin (R&D, Minneapolis, MN, USA), anti-superoxide dismutase (SOD) (Santa Cruz Biotechnology, Santa Cruz, CA, USA), anti-NOX-2, anti-NOX-4, anti-phospho-STAT-3, anti-STAT-3, anti-phospho-SMAD1/5, anti-SMAD1, anti-SMAD5, and anti-glyceraldehyde-3-phosphate dehydrogenase (GAPDH) (Cell Signaling Technology, Beverly, MA, USA). Each experiment was performed in triplicate.

### 2.9. Enzyme-Linked Immunosorbent Assay (ELISA)

Levels of hepcidin, ERFE (MyBioSource, San Diego, CA, USA), ferritin (Abcam), interleukin- (IL-) 1*β* (R&D), IL-6 (BioLegend, San Diego, CA, USA), and tumor necrosis factor-*α* (TNF-*α*) (BD Biosciences, Franklin Lakes, NJ, USA) in the serum samples were determined using ELISA commercial kits according to the manufacturer's instructions.

### 2.10. Real-Time Polymerase Chain Reaction (Real-Time PCR)

Total RNA was isolated using TRIzol reagent (Thermo Fisher Scientific). Complementary DNA was synthesised from 1 *μ*g of total RNA using *AccuPower* RT PreMix Kits (Bioneer, Daejeon, Korea). Real-time PCR analysis was performed using TB Green™ Premix Ex Taq II Kit (TaKaRa, Tokyo, Japan) and AB7500 Fast Real-Time PCR System (Applied Biosystems, Foster City, CA, USA) according to the manufacturer's instructions. Each sample was analyzed in quadruplicate, and target genes were normalized to the reference housekeeping gene *Gapdh*. Fold differences were then calculated for each group using *C*_*T*_ values normalized to those of the control groups. The sequences of all primers used are listed in Table 1.

### 2.11. Gene Knockdown Using Small Interfering RNA (siRNA)

*siRNA*s for *STAT-3*, *SMAD1*, and *SMAD5* and a nontargeting *siRNA* (control *siRNA*) were purchased from Santa Cruz Biotechnology. *STAT-3 siRNA* was a mixture of three *siRNA*s, providing advantages in terms of both potency and specificity. Transfection of *siRNA* into HepG2 cells was performed according to the manufacturer's instructions. Subsequently, the cells were incubated for an additional 24 h, with or without iron. Whole-cell lysates were prepared and subjected to immunoblotting.

### 2.12. Statistical Analyses

Data are expressed as the mean ± standard deviation (SD). Statistical analyses were performed using one-way analysis of variance and Student's *t-*test followed by Tukey correction. Statistical significance was set at *P* < 0.05. Statistical analyses were conducted using SPSS for Windows (version 25; SPSS Inc., Chicago, IL, USA).

## 3. Results

### 3.1. In ACI Rats, Hb Concentration Reduced, Whereas Inflammatory Serum Cytokine Levels Increased

**Figure 2:**
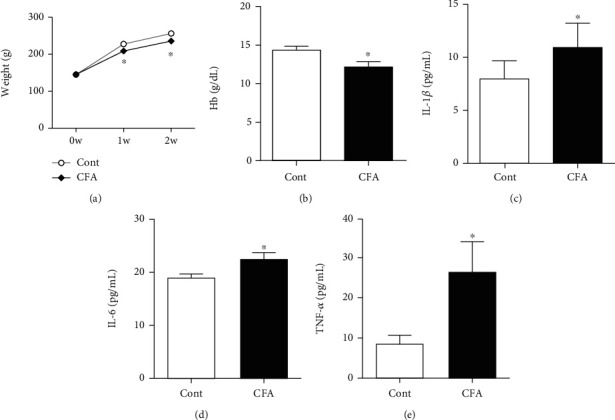
Changes in weight, hemoglobin (Hb) concentration, and serum inflammatory cytokine levels two weeks after administration of complete Freund's adjuvant (CFA). Cont: control group (*n* = 6); CFA: CFA-treated group (*n* = 6); IL-1*β*: interleukin-1*β*; IL-6: interleukin-6; TNF-*α*: tumor necrosis factor-*α*. ^∗^*P* < 0.05 compared with the control group.

CFA-treated rats (ACI rats) gained an average of 88 g, whereas rats in the control group gained an average of 102 g over the course of the two-week experimental period. The rate of weight gain in normal rats was higher than that in CFA-treated rats (Figure 2(a)). Two weeks after starting CFA treatment, Hb concentrations decreased (Figure 2(b)), whereas serum levels of inflammatory cytokines (i.e., IL-1*β*, IL-6, and TNF-*α*) significantly increased in CFA-treated rats compared with those in control rats (Figures 2(c)–2(e)).

White blood cell and neutrophil counts were increased in CFA-treated rats compared with those in control rats. The mean corpuscular volume decreased, and the red cell distribution width increased in CFA-treated rats compared with those in control rats (Table 2).

### 3.2. IV Administration of Iron Increased Hepcidin and Ferritin Levels and Decreased the ERFE Level without Changing Hb and IL-6 Concentrations in the Serum

**Figure 3:**
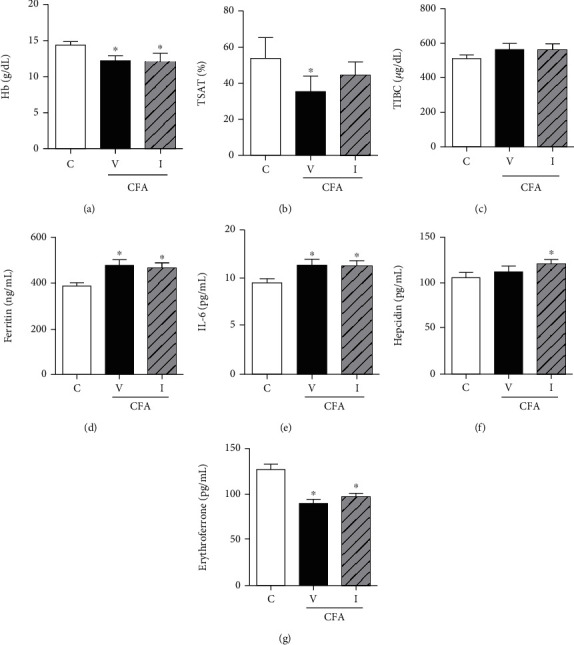
Effects of complete Freund's adjuvant (CFA) and intravenous iron treatment on the serum levels of hemoglobin (Hb), iron profiles, interleukin-6 (IL-6), hepcidin, and erythroferrone. C: control-saline group (*n* = 10); V: CFA-saline group (*n* = 10); I: CFA-iron group (*n* = 10); TSAT: transferrin saturation; TIBC: total iron-binding capacity. ^∗^*P* < 0.05 compared with the control group.

Two weeks after CFA treatment, serum levels of Hb, TSAT, and ERFE significantly decreased in the CFA-saline group compared with those in the control group (Figures 3(a), 3(b), and 3(g)), whereas serum levels of ferritin and IL-6 significantly increased in the CFA-saline group compared with those in the control group (Figures 3(d) and 3(e)). The serum hepcidin level and TIBC were similar between the CFA-saline and control groups (Figures 3(c) and 3(f)). Three days after IV iron treatment, the Hb level was still lower in the CFA-iron group than in the control group (Figure 3(a)), whereas the serum hepcidin level was significantly higher in the CFA-iron group than in the control group (Figure 3(f)). TSAT in the CFA-iron group recovered to a level similar to that in the control group (Figure 3(b)). Serum IL-6 and ferritin levels were similar between the CFA-iron and CFA-saline groups, which were significantly higher than those in the control group (Figures 3(d) and 3(e)). Serum ERFE levels were similar between CFA-iron and CFA-saline groups, which were significantly lower than those in the control group (Figure 3(g)).

### 3.3. Effects of IV Iron Supplementation in the Rat Liver

#### 3.3.1. IV Iron Supplementation Enhanced CFA-Induced Increase in Hepcidin and Ferritin Protein Levels in the Liver

**Figure 4:**
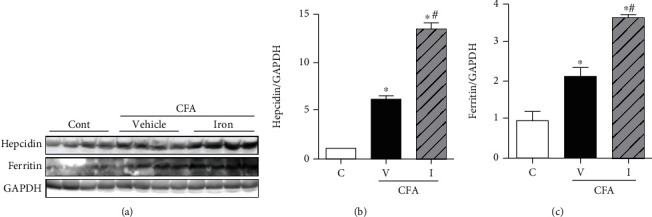
Effect of complete Freund's adjuvant (CFA) and intravenous iron treatment on the protein levels of hepcidin and ferritin in the rat liver. The relative protein levels were quantified using densitometry (lower panel). C: control-saline group (*n* = 10); V: CFA-saline group (*n* = 10); I: CFA-iron group (*n* = 10); GAPDH: glyceraldehyde-3-phosphate dehydrogenase. ^∗^*P* < 0.05 compared with the control group; ^#^*P* < 0.05 compared with the CFA-saline group.

CFA induced significant increases in the protein levels of hepcidin and ferritin in the liver of the CFA-saline group compared with those in the control group. The levels of hepcidin and ferritin were significantly increased in the CFA-iron group compared with those in the control and CFA-saline groups (Figure 4).

#### 3.3.2. IV Iron Supplementation Significantly Increased mRNA Expression of Bmp-6 and Hfe in the Liver

**Figure 5:**
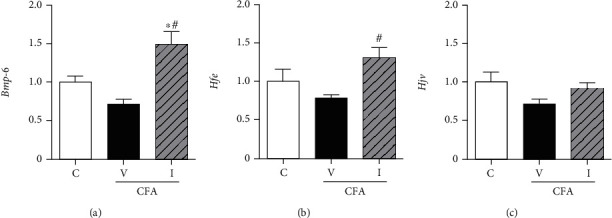
Effect of complete Freund's adjuvant (CFA) and intravenous iron treatment on the mRNA expression of bone morphogenetic protein-6 (*Bmp-6*), homeostatic iron regulator (*Hfe*), and hemojuvelin (*Hjv*) in the rat liver, as determined using real-time polymerase chain reaction. Relative transcript levels are *C*_*T*_ values normalized to those of the control group. C: control-saline group (*n* = 10); V: CFA-saline group (*n* = 10); I: CFA-iron group (*n* = 10). ^∗^*P* < 0.05 compared with the control group; ^#^*P* < 0.05 compared with the CFA-saline group.

CFA treatment did not affect the mRNA expression of *Bmp-6*, homeostatic iron regulator (*Hfe*), and hemojuvelin (*Hjv*), which are the components of the BMP/SMAD signaling pathway, in the CFA-saline rat liver (Figure 5). With IV iron supplementation, *Bmp-6*, the primary BMP associated with the regulation of hepcidin transcription via the BMP/SMAD pathway, significantly increased in the CFA-iron rat liver compared with that in the control and CFA-saline rat livers (Figure 5(a)). The mRNA expression of *Hfe*, one of the mediators of iron-mediated signaling pathways in hepatocytes, significantly increased in the CFA-iron rat liver compared with that in the CFA-saline rat liver (Figure 5(b)). The mRNA expression of *Hjv*, a coreceptor of BMP receptors, did not differ between the control, CFA-saline, and CFA-iron rat livers (Figure 5(c)).

#### 3.3.3. IV Iron Supplementation Significantly Increased Phosphorylation of Both STAT-3 and SMAD1/5 in the Liver

**Figure 6:**
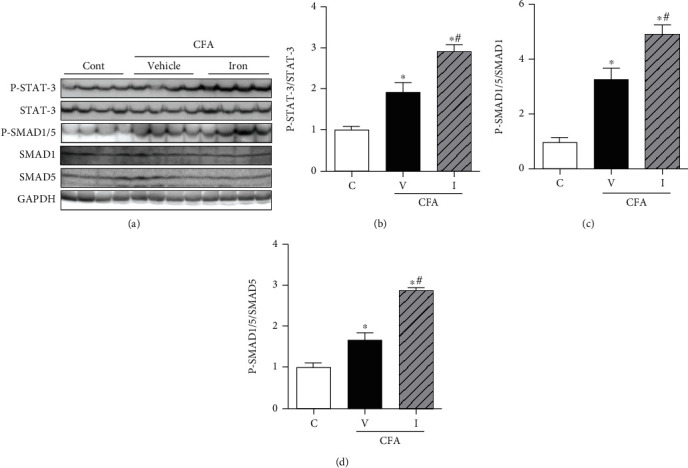
Effect of complete Freund's adjuvant (CFA) and intravenous iron treatment on the hepcidin regulation pathways in the rat liver. The relative protein levels were quantified using densitometry. C: control-saline group (*n* = 10); V: CFA-saline group (*n* = 10); I: CFA-iron group (*n* = 10); STAT-3: signal transducer and activator of transcription-3; P-STAT-3: phosphorylated STAT-3; SMAD: sma mothers against decapentaplegic; P-SMAD: phosphorylated SMAD; GAPDH: glyceraldehyde-3-phosphate dehydrogenase. ^∗^*P* < 0.05 compared with the control group; ^#^*P* < 0.05 compared with the CFA-saline group.

CFA treatment significantly increased the phosphorylation of both STAT-3 and SMAD1/5 in the CFA-saline rat liver compared with those in the control rat liver. Furthermore, IV iron supplementation significantly increased the phosphorylation of both STAT-3 and SMAD1/5 in the CFA-iron rat liver compared with those in the control and CFA-saline rat livers (Figure 6).

#### 3.3.4. IV Iron Supplementation Exacerbated CFA-Induced Increase in ROS-Generating NOX-2, NOX-4, and SOD Levels in the Liver

**Figure 7:**
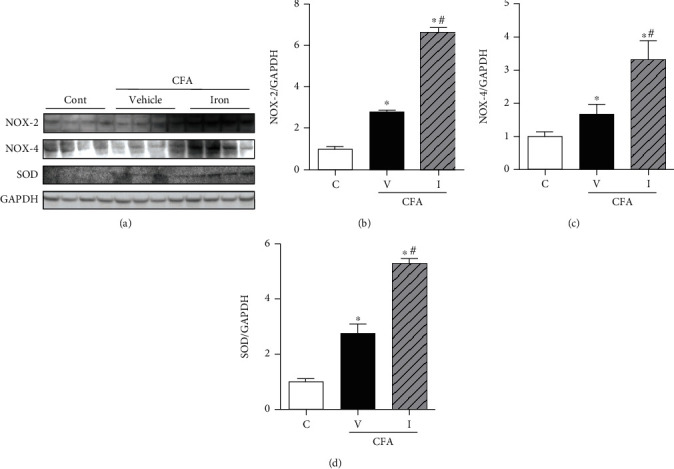
Effect of complete Freund's adjuvant (CFA) and intravenous iron treatment on the protein levels of NAPDH oxidase- (NOX-) 2, NOX-4, and superoxide dismutase (SOD) in the rat liver. The relative protein levels were quantified using densitometry. C: control-saline group (*n* = 10); V: CFA-saline group (*n* = 10); I: CFA-iron group (*n* = 10); GAPDH; glyceraldehyde-3-phosphate dehydrogenase. ^∗^*P* < 0.05 compared with the control group; ^#^*P* < 0.05 compared with the CFA-saline group.

CFA treatment significantly increased protein levels of hepatic NOX-2, NOX-4, and SOD in the CFA-saline rat liver compared with their levels in the control rat liver. The levels of these proteins further increased with IV iron supplementation in the CFA-iron rat liver compared with those in the CFA-saline rat liver (Figure 7).

**Figure 8:**
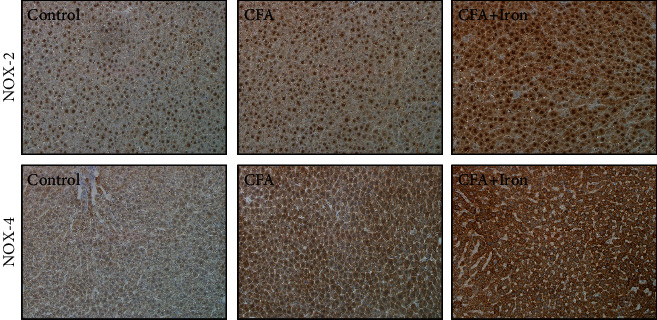
Immunohistochemistry of NAPDH oxidase- (NOX-) 2 and NOX-4 in the rat liver after complete Freund's adjuvant (CFA) and intravenous iron treatment. Control: control-saline rats (*n* = 5); CFA: CFA-saline rats (*n* = 5); CFA + Iron: CFA-iron rats (*n* = 5).

To determine whether NOX-2 and NOX-4 expression in hepatocytes was altered with IV iron supplementation, the liver specimens were subjected to IHC analyses for NOX-2 and NOX-4. The number of NOX-2- and NOX-4-positive hepatocytes was greater in the CFA-saline rat liver than that in the control rat liver. A stronger immune positivity for NOX-2 and NOX-4 was observed in the CFA-iron rat liver than those in the control and CFA-saline rat livers (Figure 8).

### 3.4. IV Iron Supplementation Significantly Reduced mRNA Expression of Epo in the Rat Kidney

**Figure 9:**
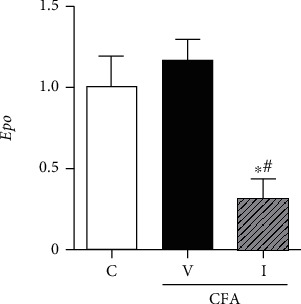
Effect of complete Freund's adjuvant (CFA) and intravenous iron treatment on the mRNA expression of erythropoietin (*Epo*) in the rat kidney, as determined using real-time polymerase chain reaction. Relative transcript levels are *C*_*T*_ values normalized to those of the control group. C: control-saline group (*n* = 10); V: CFA-saline group (*n* = 10); I: CFA-iron group (*n* = 10). ^∗^*P* < 0.05 compared with the control group; ^#^*P* < 0.05 compared with the CFA-saline group.

The mRNA expression levels of *Epo* were similar in the kidney of CFA-saline rats and control rats. IV iron supplementation significantly reduced the mRNA expression level of *Epo* in the kidney of CFA-iron rats, and these levels were significantly lower than those in the kidney of control and CFA-saline rats (Figure 9).

### 3.5. Effects of Iron Administration in HepG2 Cells

#### 3.5.1. High-Dose Iron Treatment Significantly Reduced Cell Viability in HepG2 Cells

Cell viability was measured using the CCK-8 assay after the treatment of HepG2 cells with various concentrations of iron for varying time durations. Viability of cells treated with 5–10 mg/mL of iron was significantly lower than that of control cells on both days 1 and 2 of incubation (Figure 1).

#### 3.5.2. Iron Treatment Increased Phosphorylation Levels of STAT-3 and SMAD1/5 and Protein Levels of Hepcidin and Ferritin in HepG2 Cells

**Figure 10:**
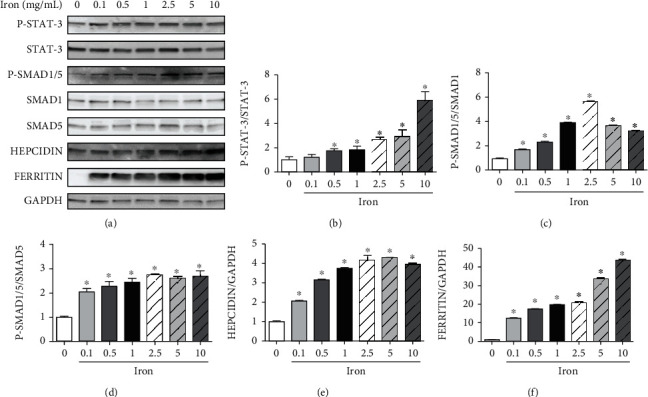
The effect of iron treatment on the phosphorylation of the signal transducer and activator of transcription-3 (STAT-3), sma mothers against decapentaplegic 1/5 (SMAD1/5), and hepcidin and ferritin protein levels in HepG2 cells. The dose of iron was increased from 0 to 10 mg/mL, and the iron-induced effects on HepG2 cells were examined using immunoblot analysis. The relative protein levels were quantified using densitometry. P-STAT-3: phosphorylated STAT-3; P-SMAD: phosphorylated SMAD; GAPDH: glyceraldehyde-3-phosphate dehydrogenase. ^∗^*P* < 0.05 compared with the control group.

The protein levels of phosphorylated STAT-3 and phosphorylated SMAD1/5 increased 24 h after iron administration in a dose-dependent manner in HepG2 cells compared with those in control cells. The protein levels of hepcidin and ferritin were subsequently increased by iron treatment in HepG2 cells compared with those in control cells (Figure 10).

#### 3.5.3. Iron-Induced Hepcidin Upregulation Involves both STAT-3 and SMAD1/5 Activation in HepG2 Cells

**Figure 11:**
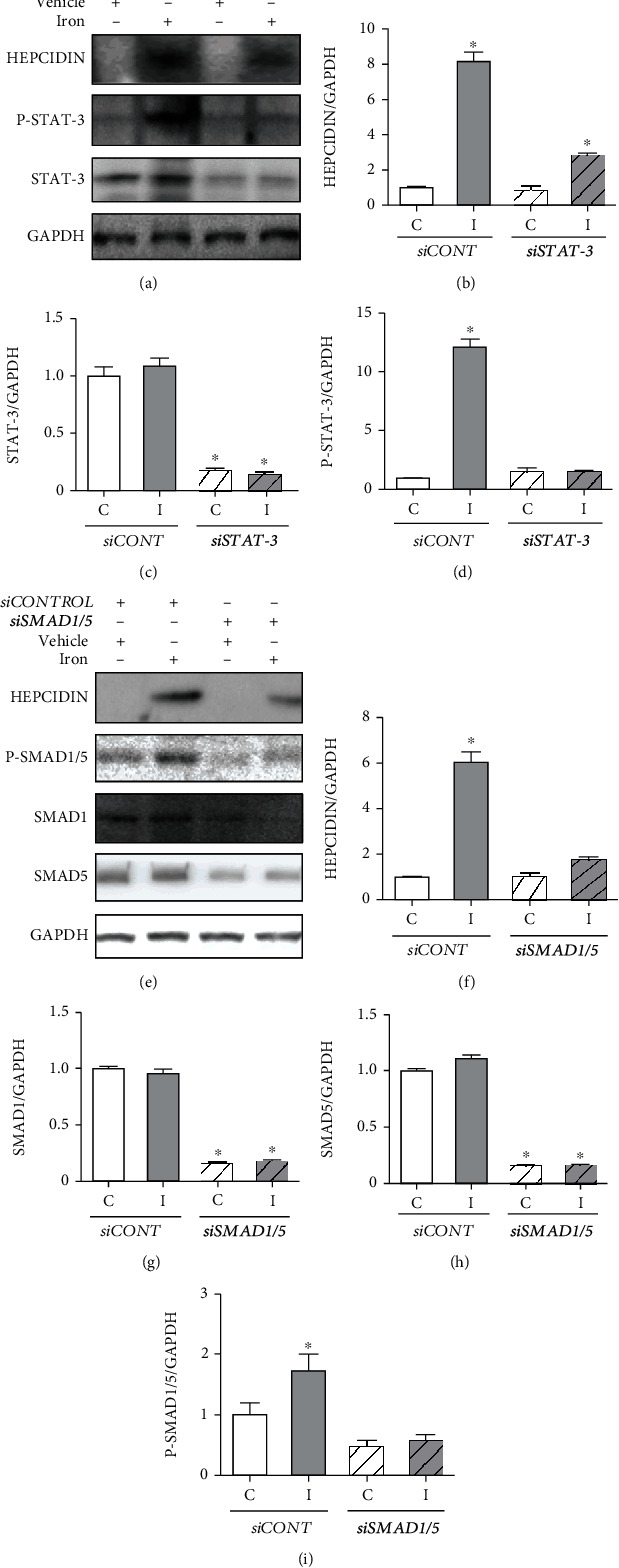
Western blot analysis was performed using HepG2 cells transfected with control small interfering RNA (*siRNA*) or *siRNA* against signal transducer and activator of transcription-3 (*STAT-3*) and sma mothers against decapentaplegic 1/5 (*SMAD1/5*) to investigate hepcidin expression after iron treatment (2.5 mg/mL). The relative protein levels were quantified using densitometry (b–d and f–i). *siCONTROL*: control *siRNA*; *siSTAT-3*: *siRNA* against *STAT-3*; P-STAT-3: phosphorylated STAT-3; *siSMAD1/5*: *siRNA* against *SMAD1/5*; P-SMAD1/5: phosphorylated SMAD1/5; GAPDH: glyceraldehyde-3-phosphate dehydrogenase; C: control group; I: iron-treated group. ^∗^*P* < 0.05 compared with the control group.

To further define the function of STAT-3 and SMAD1/5 in iron-mediated regulation of hepcidin, we performed *siRNA*-mediated knockdown of endogenous *STAT-3* and *SMAD1/5*. The efficacy of *siRNA*-mediated knockdown was verified using immunoblotting (Figures 11(a) and 11(e)). Iron treatment led to an increase in hepcidin levels in control HepG2 cells, and knockdown of *STAT-3* and *SMAD1/5* reduced the iron-induced upregulation of hepcidin expression. Hepcidin expression in HepG2 cells treated with *siSTAT-3* was still significantly greater after iron treatment than that in the control group, whereas its expression in cells treated with *siSMAD1/5* was comparable between the groups. These results suggest that iron-mediated upregulation of hepcidin involves activation of STAT-3 and SMAD1/5 (Figure 11).

## 4. Discussion

In clinical practice, preoperative IV iron supplementation is advocated in anemia patients with heart failure and/or those undergoing cardiac or oncologic surgeries in whom ACI frequently occurs, with the aim of improving the patients' outcome related to anemia and transfusion [21, 22]. However, by far, the available evidence is conflicting [23]. Although it has been assumed that hepcidin, a key regulator of iron homeostasis, may hinder effective iron utilization for erythropoiesis when upregulated by external iron supplementation and systemic inflammation, little is known about the changes in the expression of hepcidin after a large bolus of IV iron administration in patients with ACI. In this context, signaling pathways associated with hepcidin regulation and erythropoiesis after IV iron supplementation were examined in the current study, specially focused on the changes on day three because the short-term treatment effect of a high-dose IV iron supplementation has been a concern in patients undergoing surgery [15, 16].

In this study, we utilized a rat model of ACI with CFA treatment that resembled the clinical presentations of ACI, involving anemia accompanied by iron deficiency and chronic systemic inflammation. Three days after IV iron supplementation at a clinically relevant dose, the Hb level did not increase, whereas hepcidin activity was upregulated along with increases in the phosphorylated levels of not only SMAD1/5 but also STAT-3 in CFA-treated rats. Concurrent hepatic upregulation of NOX-2, NOX-4, and SOD protein levels following IV iron supplementation is likely to implicate a potential link between iron-induced oxidative stress and the activation of STAT-3 because IL-6 levels did not change after IV iron supplementation in CFA-treated rats. IV iron supplementation significantly reduced renal *Epo* mRNA expression and the serum ERFE level in CFA-treated rats compared with those in control rats.

### 4.1. IV Iron and Hepcidin in the ACI Rat Model

To conduct the study under conditions as close as possible to clinical ACI, the experiments were carried out in rats that had been subjected to moderate, repeated inflammatory stimuli through IP-administered CFA across two weeks, unlike previous studies that conducted experiments after inducing inflammation with a single IP administration of pseudoinfectious particles (e.g., CFA) [20, 24, 25]. The ACI state in CFA-treated rats could be verified by decreased Hb concentrations and serum levels of TSAT and increased leukocyte counts and inflammatory cytokine levels, including IL-1*β*, IL-6, and TNF-*α*, compared with those in control rats at two weeks after starting CFA treatment.

Hepcidin expression is regulated by the IL-6/STAT and BMP/SMAD signaling cascades in the liver [4, 5]. In many models of anemia of inflammation, IL-6 has been shown to be a primary driver for hepcidin induction and the SMAD1/5/8 pathway is also known to contribute, likely via activin B and STAT-3-SMAD interactions at the hepcidin promoter [26]. Similarly, herein, in the CFA-saline rat livers, phosphorylation of both STAT-3 and SMAD1/5 proteins significantly increased along with elevated serum IL-6 levels compared to those in control rats, whereas mRNA expression of *Bmp-6*, *Hfe*, and *Hjv* was comparable to those in the control rat livers. Additionally, markers of oxidative stress, such as NOX-2, NOX-4, and SOD, significantly increased in the CFA-saline rat livers compared to those in the control rat livers.

Importantly, IV iron supplementation resulted in significantly higher hepatic hepcidin mRNA (data not shown) and protein levels in CFA-iron rats than those in CFA-saline rats, whereas serum IL-6 levels were similar. In this regard, the activities of STAT-3 as well as BMP/SMAD pathways and markers of oxidative stress were significantly augmented in the CFA-iron rat livers compared with those in the CFA-saline rat livers. Moreover, the mRNA expression of *Hfe* that acts as an iron sensor and upstream regulator of hepcidin transcription [27] was intensified with IV iron supplementation in the CFA-iron rat livers compared with that in the CFA-saline rat livers.

### 4.2. IV Iron and Oxidative Stress

The link between IV iron and STAT-3 activity was established in cell experiments. In HepG2 cells transfected with *siSTAT-3*, hepcidin upregulation by iron administration was significantly attenuated compared with that in the nontransfected cells, indicating a direct association between iron load and STAT-3 activity.

IV iron has been known to cause oxidative stress, and STAT-3 is involved in both inflammation and ROS-mediated hepcidin regulation, mainly involving IL-6 and NOX-4 [28, 29]. NOX-4 is a member of a family of enzymes comprising seven isoforms responsible for producing various ROS. Of these family members, two isoforms—NOX-2 and NOX-4—have been previously demonstrated to be powerful inducers of hepcidin expression in the liver via the STAT-3 signaling pathway [30]. Simultaneous increase in NOX-2, NOX-4, and STAT-3 activities after IV iron supplementation in this study is consistent with the results of previous studies. These findings imply a potential relationship between ROS-generating NOX-2, NOX-4, and SOD and an additional increase in hepcidin expression after IV iron supplementation, although we did not investigate whether simultaneous treatment with antioxidant and IV iron could attenuate the activation of STAT-3 and subsequent elevation of hepcidin in this study. Importantly, IV iron-induced oxidative stresses have been reported to depend on various factors that include not only complex stability but also carbohydrate ligand and particle size [31]. The fact that nontransferrin binding iron (labile iron) is not the only possible source of iron-induced oxidative stress can warn against the liberal use of high doses of stable complexes like iron isomaltoside used in the present study [32].

### 4.3. IV Iron Supplementation and Erythropoiesis in ACI Rats

In alignment with several clinical studies, in this study, Hb did not increase three days after IV iron supplementation in CFA-iron rats compared with that in CFA-saline rats. In humans, erythropoiesis is known to increase 5–7 days after IV iron supplementation. Considering that the lifespan of erythrocytes in humans is about twice that of rats [33, 34], a duration of 2–4 days to activate erythropoiesis was expected in response to a large IV iron load in rats and we assessed the results three days after IV iron supplementation in CFA-treated rats. A substantial increase in hepcidin levels in response to IV iron could have potentially sequestered iron as ferritin in the storage cells (e.g., hepatocytes), as shown in the current study, thereby impeding iron utilization for erythropoiesis and hindering Hb increase. Increased oxidative stresses that were further enhanced after IV iron treatment would have also corroborated with the negative erythropoietic effect of IV iron on ACI rats as seen in this study.

### 4.4. IV Iron and EPO Regulation in ACI Rats

In the present study, serum ERFE levels decreased in CFA-treated rats compared with those in control rats. Serum ERFE is produced by EPO-stimulated erythroblasts during erythropoiesis, and this result is possibly attributable to a blunted response to EPO under chronic inflammatory conditions [35]. Additionally, IV iron supplementation significantly diminished renal *Epo* mRNA expression in CFA-iron rats compared with those in control and CFA-saline rats. *EPO* transcription in the kidney is controlled by hypoxia-inducible transcription factor 2*α* (HIF2*α*). Oxygen and iron are well-known factors that determine the activity of HIF2*α* through HIF-prolyl hydroxylase- (PHD-) induced degradation [36]. In this context, IV iron supplementation could have caused an increase in PHD activity, leading to subsequent decreases in HIF2*α* and *Epo* mRNA expression [37], although we did not evaluate the corresponding signaling pathways in this study. Oxidative stress-dependent HIF2*α* inactivation was also reported as a possible mechanism of iron-induced suppression of renal *EPO* expression [38], and increased oxidative stress markers in the present study might be related with a reduced mRNA expression of renal *Epo* in CFA-iron rats. Considering that erythropoiesis-stimulating agent inhibits hepcidin expression through ERFE [39, 40], these changes might also have contributed to enhanced hepcidin activity. Based on these results, we can assume that the simultaneous regulation of both hepcidin and EPO is required to induce effective erythropoiesis after IV iron supplementation in a clinically relevant short period in patients with ACI.

### 4.5. Limitations

This study has the following limitations. First, although the ACI rat model used in this study seems to be clinically relevant, we focused on the short-term effect of IV iron supplementation. Anticipating that its effects on hepcidin and erythropoiesis would be dynamic, further studies are warranted to investigate the response of hepcidin and its regulatory pathways to IV iron supplementation across a prolonged period. Second, the link between oxidative stress and hepcidin expression and the link between renal HIF2*α* and EPO expression in association with IV iron supplementation in ACI rats were not directly verified in the current study, although they have been reported previously under various conditions.

## 5. Conclusions

Hepatic hepcidin protein activity was upregulated in CFA-treated rats, and IV iron supplementation at a clinically relevant dose intensified the degree of hepatic hepcidin upregulation. Three days after IV iron treatment of CFA-iron rats, Hb levels did not change, despite the increase in liver ferritin levels, and renal *Epo* mRNA expression was reduced. Both STAT-3 and SMAD1/5 were identified as relevant pathways of hepatic hepcidin upregulation in relation to IV iron supplementation in CFA-iron rats. STAT-3 activation in the absence of IL-6 increase may be associated with the IV iron-induced increase in oxidative stress, indicated by an increase in hepatic NOX-2 and NOX-4 activity. The current study provides primary evidence regarding the need for simultaneous regulation of hepcidin and EPO to enhance the efficacy of IV iron supplementation for the treatment of anemia under chronic inflammatory conditions.

## Figures and Tables

**Table 1:** Sequences of the primers used for real-time polymerase chain reaction.

Symbol	Accession number	Primers		Full name
*Bmp-6*	NM_013107.1	Forward	5′-GCTCCAGTGCTTCAGACTAC-3′	Bone morphogenetic protein-6
		Reverse	5′-GATGATCCAGTCCTGCCATC-3′	
*Hfe*	XM_032884765	Forward	5′-AGATGCCAAGGATGTCAACC-3′	Homeostatic iron regulator
		Reverse	5′-TCTTGTCTCTTCTCCAGGGG-3′	
*Hjv*	NM_001012080	Forward	5′-TGCAGCCTTTGAAGATGGTT-3′	Hemojuvelin
		Reverse	5′-TGTTCCAATGTAGGCAGCTC-3′	
*Epo*	NM_017001	Forward	5′-CTATTTACGGGGTGCTGGAC-3′	Erythropoietin
		Reverse	5′-ATGAGTTTGGCTGTCTCTGC-3′	
*Gapdh*	NM_017008	Forward	5′-AACGACCCCTTCATTGACCT-3′	Glyceraldehyde-3-phosphate dehydrogenase
		Reverse	5′-TGACCAGCTTCCCATTCTCA-3′	

**Table 2:** Leukocyte counts and red cell indices after complete Freund's adjuvant (CFA) treatment.

	Control rats (*n* = 6)	CFA-treated rats (*n* = 6)
White blood cells (×10^3^/*μ*L)	5.6 ± 0.3	9.9 ± 1.2^∗^
Neutrophil (×10^3^/*μ*L)	1.17 ± 0.1	3.80 ± 0.7^∗^
Lymphocyte (×10^3^/*μ*L)	4.0 ± 0.2	5.2 ± 0.8
MCV (fL)	68.3 ± 0.4	66.9 ± 0.7^∗^
MCH (pg)	22.7 ± 0.2	22.2 ± 0.3
MCHC (g/dL)	33.2 ± 0.3	33.2 ± 0.2
RDW (%)	12.4 ± 0.1	15.9 ± 0.4^∗^

The data are presented as the mean ± SD. MCV: mean corpuscular volume; MCH: mean corpuscular hemoglobin; MCHC: mean corpuscular hemoglobin concentration; RDW: red cell distribution width. ^∗^*P* < 0.05 compared with the control group.

## Data Availability

The data used to support the findings of this study are available from the corresponding authors upon request.
